# An Estimation of the Prevalence and Progression of Chronic Kidney Disease in a Rural Diabetic Cambodian Population

**DOI:** 10.1371/journal.pone.0086123

**Published:** 2014-01-22

**Authors:** Bernadette Thomas, Maurits van Pelt, Rajnish Mehrotra, Cassianne Robinson-Cohen, James LoGerfo

**Affiliations:** 1 Division of Nephrology, Department of Medicine, University of Washington, Seattle, Washington, United States of America; 2 MoPoTsyo Patient Information Centre, Phnom Penh, Cambodia; 3 Kidney Research Institute, University of Washington, Seattle, Washington, United States of America; 4 Department of Medicine, University of Washington, Seattle, Washington, United States of America; University of Leicester, United Kingdom

## Abstract

**Background:**

To date, there are no known estimates of the prevalence of chronic kidney disease within Cambodia, the vast majority of whose citizens live in rural areas with limited access to renal replacement therapy.

**Methods:**

Observational analysis of patients from the Takeo province in Cambodia who presented to MoPoTsyo, a non-governmental organization, for screening and management of diabetes mellitus between 2010 and 2012 (n = 402; 75% females). Estimated glomerular filtration rate (eGFR) was calculated using the CKD-Epi equation.

**Results:**

On average, women were younger, with a higher percentage of hypercholesterolemia but also high-density lipoprotein level. Men had a higher serum creatinine level (1.31 mg/dl) than that of women (1.13 mg/dl) at 95% CI. More than half of all screened patients had a reduced eGFR; **60**% (95% CI 55%, 65%) had an eGFR<60 ml/min/1.73 m^2^; **54%** (49%, 59%) had an eGFR 30–60 ml/min/1.73 m^2^, and **5.7%** (3.4%, 8.0%) with eGFR 15–30 ml/min/1.73 m^2^. Women had a greater prevalence of **stage 3** CKD (57% women vs. 47% men) and **stage 4** CKD (7.0% vs. 2.0%). The adjusted odds ratio for females compared to males having an eGFR <60 ml/min/1.73 m^2^ was **3.19** (95% CI 1.78, 5.43; p value<0.001). Thirty-two percent of patients lost ≥5 ml/min/1.73 m2 eGFR during median follow-up time of 433 days (IQR 462 days) days.

**Conclusions:**

Over one-half of Cambodians with diabetes mellitus had reduced eGFR, implying a point-prevalence of chronic kidney disease of 1.2% in among adult Cambodians within the country. This high burden of kidney disease in a society that lacks universal access to renal replacement therapy underscores the importance of early diagnosis – a largely unmet need in Cambodia.

## Introduction

The projected rise in global rates of diabetes mellitus and hypertension portend increasing rates of associated diseases such as chronic kidney disease (CKD) [1]–[6]. Resource-limited countries lacking the ability to offer renal replacement therapy for end-stage renal disease (ESRD) face the significant challenge of early kidney disease detection to allow for timely intervention to retard disease progression [1], [2], [6], [7]. Yet in many regions of the world, little data exist estimating the prevalence of CKD [8]–[11].

The south-east Asian kingdom of Cambodia lies bordered between Vietnam, Thailand, Laos, and the Gulf of Thailand. In 2010, the country population was totaled to be 14.1 million, with a life expectancy of 64.6 years for men and 70.1 years for women.^12^ The World Health Organization lists Cambodia as a low-income country, with about 80% of its inhabitants living in rural settings. It is estimated that about 46% of all deaths in the country are secondary to non-communicable diseases [12].

In 2010, a national cross-sectional STEPS survey was performed in Cambodia estimating the prevalence of non-communicable diseases, allowing for the first-ever estimation of conditions that increase the risk for CKD. According to study results, about one in every ten respondents had hypertension and 2.9% of individuals were diagnosed with diabetes mellitus [13]. The prevalence of both hypertension and diabetes were significantly more common in the urban than in the rural areas. The results of the study also described the relationship between gender and chronic conditions. Hypertension was more frequent in men, but women had a higher prevalence of elevated total cholesterol and obesity [13].

These data raise concern for a large undetected burden of kidney disease in the country. However, to date no estimates on the prevalence of reduced renal function – either in the population-at-large or in high-risk sub-groups with diabetes and/or hypertension – have been made from anywhere in Cambodia. In order to bridge this gap in our knowledge, we undertook this analysis to develop the first-ever estimates of the prevalence of reduced glomerular filtration rate (GFR) in a screened cohort of individuals with diabetes mellitus from a rural community in Cambodia with a specific focus on gender differences in the prevalence of diabetic CKD.

## Materials and Methods

This analysis was exempt from review by the Institutional Review Board of the University of Washington as it describes results of data collected for program evaluation.

This population-based analysis of renal function in adults from the Takeo province of southern Cambodia was performed using screening data gathered between 2010 and 2012 by the non-profit organization MoPoTsyo.

### Data Source

MoPoTsyo is a Cambodian organization founded in 2004 to screen inhabitants of resource-limited regions for diabetes mellitus and hypertension [14]. The organization uses a peer-education network system to achieve its objectives of education, screening, and treatment for chronic diseases [14]. Analysis of data from Takeo province was selected due to the completeness of data over a two-year period. All patients included in this analysis had an existing diagnosis of diabetes mellitus at time of entry into MoPoTsyo, or were diagnosed to be diabetic by this non-governmental organization.

### Data Collection

Individuals from the community were invited to present for screening or evaluation of previously diagnosed diabetes mellitus. Initial screening and data-gathering was performed by Cambodian peer educators trained by MoPoTsyo. The initial evaluation involved collection of demographic, lifestyle and diet information, exercise habits, height, weight, waist, and hip circumference, and current medications. The height and weight data were used to calculate the body mass index (BMI; kg/m^2^). A fasting sample of blood was drawn for the measurement of baseline serum creatinine, cholesterol, and liver function measurements. Patients without a pre-existing diagnosis of hypertension were diagnosed to be hypertensive if their initial blood pressure was greater than 130/80 mmHg.

A total of 440 individuals with diabetes mellitus presented for the initial screening. However, data on eGFR were unavailable for 38 individuals, yielding an analytic cohort of 402 patients. Medication data were available for 366 of the 402 patients included in this analysis. A total of 398 individuals had eGFR re-evaluated after a median of 433 days (interquartile range 462 days).

### Laboratory measurement

Serum creatinine was measured using the kinetic rate Jaffe method, and was aligned using isotope dilution mass-spectrometry (IDMS). Estimated GFR (eGFR) was calculated using the CKD-Epi equation based on measured serum creatinine. The CKD-Epi equation was chosen as it has been assessed to be more accurate than the Modification of Diet in Renal Disease (MDRD) equation in both high and low risk CKD populations [15].

Patients were grouped into stages of CKD, as defined by the National Kidney Foundation Kidney Disease Outcomes Quality Initiative (KDOQI): stage 3, eGFR, 30–60 ml/min/1.73 m^2^; stage 4, 15–30 mlmin/1.73 m^2^, and stage 5, <15 ml/min/1.73 m^2^ or undergoing renal replacement therapy.

### Statistical Analysis

Patients' age, BMI, waist circumference, waist-to-hip ratio, blood pressure, total cholesterol, and medication use are presented as mean values with 95% confidence intervals, stratified by gender (Tables 1, 2). Student's t-tests and Chi-square tests were performed to determine the significance of difference, by gender, evaluate of continuous and categorical characteristics, respectively, as listed in Tables 1 and 2. Hypercholesterolemia was defined as a serum total cholesterol level ≥240 mg/dl [16]. Uncontrolled hypertension was defined as a blood pressure recording ≥140/90 mmHg.

**Table 1:** Patient demographics.

	Male	Female	Total
	100	302	402
Age, years*	56.1 (53.9, 58.2)	53.6 (52.4, 54.7)	54.2 (53.2, 55.1)
Body Mass Index (kg/m^2^)	23.7 (23.0, 24.4)	23.2 (22.8, 23.6)	23.3 (23.0, 23.7)
Waist circumference (cm)	85.5 (83.4, 87.6)	83.4 (82.2, 84.6)	83.9 (82.9, 85.0)
Waist-Hip Ratio	0.9 (0.9, 0.9)	0.9 (0.9, 0.9)	0.9 (0.9, 0.9)

Results indicate mean values unless otherwise indicated.

Parentheses indicate 95% confidence intervals.

* indicates a statistically significant association with gender (p value<0.05).

**Table 2:** Disease and medication history.

	Male	Female	Total
	100	402	302
**Diabetes Mellitus**			
Time since diagnosis (years, mean)	4.8 (4.2, 5.3)	5.0 (4.7, 5.3)	4.95 (4.7, 5.2)
Fasting blood glucose (mg/dl; mean)	187.8 (174.5, 201.1)	191.6 (183.2, 199.9)	190.6 (183.5, 197.7)
Post-prandial glucose (mg/dl; mean)	284.2 (261.9, 306.4)	284.8 (273.4, 296.2)	284.7 (274.5, 294.8)
**Blood Pressure** (mmHg), mean			
Systolic (mmHg)^a^	132 (127, 137)	133 (130, 136)	133 (130, 135)
Diastolic (mmHg)^a^	83 (80, 85)	84 (83, 85)	84 (82, 85)
Established diagnosis of Hypertension (mean)*	13	23	20
**Cholesterol (mg/dl) mean**			
Total cholesterol ^b^	169 (158, 181)	168 (161, 176)	169 (162, 175)
HDL* ^c^	41 (38, 45)	46 (44, 49)	45 (43, 47)
Hypercholesterolemia %	7	12	10.7
Triglycerides	243 (214.4, 272.8)	270.1 (251.4, 288.9)	263.5 (247.7, 279.4)
**Medications ^d^** (mean)			
Ace inhibitor	42.4 (36.3, 48.4)	44.2 (34.5, 53.9)	42.8 (37.7, 47.9)
Diabetic medications	74 (65, 83)	85 (80, 89)	82 (78, 86)

Abbreviation: HDL, high density lipoprotein.

Results indicate mean values unless otherwise indicated.

Parentheses indicate 95% confidence intervals.

* indicates a statistically significant association with gender (p value<0.05).

a missing: 1 male, 1 female; ^b^ missing: 3 male, 16 female; ^c^ missing: 19 male, 67 female; ^d^ among 366 patients.

Blood pressure and blood sugar were assessed by MoPoTsyo staff who were trained to take these measurements using automated sphygmomanometers and handheld glucometers.

**Figure 1:**
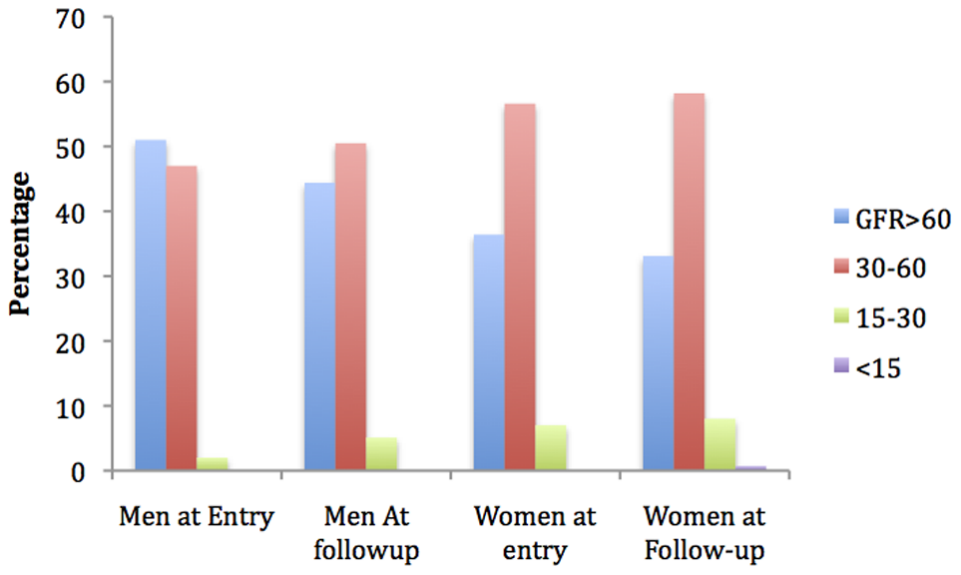
Renal function at entry and follow-up stratified by gender.

Change of renal function over time was assessed by calculating the mean percentage of patients who lost between 5–10, 10–15, and >15 ml/min/1.73 m^2^ of eGFR between initial screening and follow-up (Figure 1). Statistical significance for outcome stratified by gender were determined using chi square analyses, with significance being interpreted as a p value<0.05.

Logistic regression was performed to examine the association between gender and reduced eGFR as defined by an eGFR ≤60 mmin/1.73 m^2^. Unadjusted and adjusted results for available Framingham risk factors of age, BMI, blood pressure, total cholesterol, and high-density (HDL) cholesterol we calculated.

All analyses were performed using a licensed version of Stata 12.1. Values were considered statistically significant at the α = 0.05 level.

## Results

### Patient characteristics and biological profile

The majority of patients that presented for screening were female. On average, women were younger and had higher total cholesterol levels, but also slightly higher HDL cholesterol (see Table 1, 2). There was no significant difference in mean blood pressure or BMI measurements between genders (Table 2).

Women were more likely to be taking or be prescribed treatment for high blood pressure, diabetes, or hypercholesterolemia at time of screening than men (Table 2).

### Estimated glomerular filtration rate

On average, men had higher serum creatinine than women. While only 1.7% of women had serum creatinine >2 mg/dl, 7% of men had a serum creatinine value greater than 2 mg/dl (Table 3; (p-value<0.05).

**Table 3:** Creatinine in patients at entry.

Creatinine (mg/dl)	Male	Female	Total
	n = 100	n = 302	n = 401
< = 1mg/dl*	16 (9, 23)	48 (42, 53)	39.8 (35, 45)
1–2 mg/dl*	77 (69, 85)	51 (45, 56)	57 (52, 62)
2–3 mg/dl*	7 (2, 12)	1.3 (0.03, 2.6)	3 (1, 4)
>3mg/dl	0.0 (0.0, 0.0)	0.3 (−0.3, 1.0)	0.2 (−0.2, 0.7)

Results indicate percentages.

Parentheses indicate 95% confidence intervals.

* indicates a statistically significant association with gender (p value<0.05).

A total of 60% (N) of patients had an eGFR less than 60 ml/min/1.73 m^2^. Women had a higher CKD prevalence per category of CKD for both stages 4 and 5, though this difference was not statistically significant (Table 4).

**Table 4:** Estimated glomerular filtration rate in patients at entry.

GFR^a^ (ml/min/1.73m^2^)	Male	Female	Total
	n = 100	n = 302	n = 402
>60*	51 (41, 61)	36 (31, 42)	40 (35, 45)
30–60	47 (37, 57)	57 (51, 62)	54 (49, 59)
15–30	2 (0, 5)	7 (4, 10)	6 (3, 8)
<15	0.0	0.0	0.0

Abbreviation: GFR, glomerular filtration rate.

Results indicate percentages.

Parentheses indicate 95% confidence interval.

* indicates a statistically significant association with gender (p value<0.05).

### Renal Function at Follow-Up

Upon follow-up, (20) 20.2% of men and (50) 16.7% of women with eGFR >60 ml/min/1.73 m^2^ at baseline were determined to have an eGFR of <60 ml/min/m^2^ (Tables 4 and 5, Figure 1).

**Table 5:** Estimated glomerular filtration rate in patients at follow-up.

GFR (ml/min/1.73m^2^)	Male	Female	Total
	n = 99	n = 299	n = 398
>60*	44 (35, 54)	33 (28, 39)	36 (31, 41)
30–60	51 (41, 61)	58 (53, 64)	56 (51, 61)
15–30	5 (1, 9)	8 (5, 11)	7 (5, 10)
<15	0.0 (0,0)	1 (0, 1.6)	0.5 (−.2, 1)

Abbreviation: GFR, glomerular filtration rate.

Results indicate percentage.

Percentages indicate 95% confidence interval.

* indicates a statistically significant association with gender (p value<0.05).

### Gender and CKD Progression

Of the 398 patients who had a second creatinine reading taken within a median of 433 days, 32% of patients lost ≥5 ml/min/m^2^ of eGFR, 24% lost greater than 10 ml/min/m^2^, and 13% lost greater than 15 ml/min/m^2^. The difference between women (32.6%, 95% CI: 0.27, 0.38) and men (31.3%; 95% CI 0.22, 0.41) who lost ≥5 ml/min/1.73 m^2^ of eGFR was not determined to be statistically significant (p value 0.82) (Table 5, Figure 1).

### Gender and CKD Prevalence

The unadjusted odds ratio (OR) of the association between female gender and an impaired eGFR at initial assessment was 1.82 (95% CI 1.15, 2.87), with an adjusted OR of 3.19 (95% CI 1.72, 5.93, p value<0.001). Analysis was adjusted for available Framingham risk factors of age, body mass index, systolic blood pressure, diastolic blood pressure, total cholesterol, and high-density lipoprotein (HDL) cholesterol.

### Estimate of population size with reduced eGFR

We performed a crude estimation of the percentage of diabetics within the Cambodian population calculated to have a reduced eGFR. The 2010 Cambodian census lists the national population at 14.1 million, 60.5% of which were between the ages of 25–64 years [17]. 2.9% of the population between the ages of 25–64 years was determined to have elevated blood glucose according to the 2010 Cambodian STEPS survey. Of those individuals, from our results we estimate 40.0% to have a reduced eGFR if this cohort is similar to other diabetics in Cambodia. This would amount to at least 98,954 adult individuals with diabetes mellitus and an abnormal eGFR, or 1.2% of the Cambodian population between ages 25–64 years.

## Discussion

To our knowledge, this is the first-ever report of the prevalence of reduced eGFR from population-based screening within Cambodia. The results of this study involving about 400 rural diabetic Cambodian men and women indicate that there is a high prevalence of reduced renal function within this high-risk population. Reduced renal function was found to be more prevalent in women, and despite treatment of blood pressure and diabetes, kidney disease progression was noted in a significant proportion of individuals of both genders. Male patients had a lower prevalence of reduced renal function at entry, but a higher percentage progressed from normal kidney function to a diseased state. Very few men or women were found to be in or progress to advanced renal insufficiency. Our results estimate the percentage of the national diabetic population with advanced renal insufficiency to be 1.2%; this assessment does not include individuals with significant albuminuria and preserved eGFR as albuminuria data was not systematically collected for all patients. Hence, the true percentage of Cambodians with reduced renal function can be assumed to be significantly higher secondary to unmeasured earlier stages of CKD as diagnosed by persisting albuminuria with preserved eGFR.

There is expansive literature describing the role of gender in population access to primary care services, describing that women are more likely to access both mental health and primary care services than men [18]–[20]. The 2010 Cambodian STEPS survey and 2010 Cambodia Demographic and Health Survey results indicate this pattern to be similar within Cambodia. The 2010 STEPS study published results describing a higher initial inclusion of women than men secondary to responsiveness, and a higher response of women than men during each of the three steps of the evaluation. Further, the results of two large population-based screening studies among adults in Cambodia for diabetes and associated diseases also involved unequal gender responsiveness for unclear reasons [21]. The results of our study also indicate a noted predominance of women seeking screening or assistance with management of previously diagnosed chronic diseases. Though this gender predominance limits the ultimate generalizability of study results to the national Cambodian population, the value in these first estimates of kidney function warrant crude estimation of population burden of kidney disease until more exact estimates are available through studies structured to include more balanced gender inclusion.

The Cambodian STEPS survey also indicates that 2.3% of rural respondents and 5.6% of urban respondents had elevated fasting glucose or diabetes, a greater percentage of which were female [13]. There is a clear difference in burden of disease based on geography and gender; thus further studies are needed to more accurately characterize the incidence and progression of chronic kidney disease in both rural and urban settings. Notable differences between the MoPoTsyo participants and Cambodian STEPS study participants are that the STEPS study includes a wider age range, an urban population and larger sample size. Thus the extrapolation of national estimates of advanced renal insufficiency based on national STEPS survey report are rough estimates at best. Also, the differences in prevalence and progression of renal insufficiency by gender identified in this study will need to be validated with a larger cohort with more even distribution of men to women.

Data on the population prevalence of reduced renal function in southeast Asian countries are limited [2], [7], [11], [22]. A recent study performed in northern Vietnam, involving about 8,500 adults, detected 3.1% of participants to have CKD stage 3–5 with use of the Cockcroft-Gault equation, 1.9% with use of the MDRD equation, and 3.6% when the MDRD equation was used with adjustment using the Japanese coefficient [7]. We note the wide heterogeneity of estimation depending on the GFR estimation equation [7]. In contrast, a recent publication from India detailing the first estimates from the country's renal registry revealed almost 50% of presenting cases to be CKD stage 5 [22]. This report from India emphasizes the importance of screening programs in resource-poor countries like Cambodia that lack a healthcare system that could bear the significant financial burden of universal access to maintenance renal replacement therapy; in such countries, many of the individuals who present with late-stage CKD are likely to die from the consequences of kidney disease.

As chronic diseases continue to play a larger role in early mortality within developing populations, accurate estimates of population burden of chronic diseases will help to determine the true population at risk. Slowing progression to ESRD is a major impetus for CKD detection in populations with limited ability to offer long-term renal replacement therapies such as dialysis and transplantation [3], [4]. Early detection offers the possibility of implementation of treatments that can retard progression [4]. Early-stage detection has the added importance of understanding the mortality-risk of the CKD population, as CKD is associated with an increased likelihood of mortality secondary to cardiovascular disease, as well as being an independent risk factor for early mortality prior to development of ESRD [23].

The limitations of our study include its sample size, that all patients reside in rural areas of Cambodia, and that the CKD-Epi equation used to quantify renal function has not been validated for the Cambodian population. Further, women were over-represented in the cohort, and as we did not have urinalyses for all patients, we were limited in our ability to assess earlier stages of renal insufficiency. The strengths of this study are that it is the first to our knowledge to quantify kidney function estimates for a high-risk population within Cambodia.

Studies will need to be performed to assess the burden of disease in urban populations. Future studies will also be needed to explore the association between gender and chronic kidney disease both in rural and urban settings. Inclusion of kidney function in future STEPS analyses would greatly assist in enumerating the burden of disease within the country as well as within high-risk groups such as diabetics and patients with hypertension. National or region-level renal registries would also direct preventive measures for reducing the incidence and progression of CKD, and help target how and where best to implement renal replacement therapies. Ultimately, a multi-tiered approach of screening, disease treatment, and renal replacement therapy program development will be necessary to address the anticipated increase in the number of Cambodian individuals with CKD.
